# Clinical manifestations, antimicrobial resistance and genomic feature analysis of multidrug-resistant *Elizabethkingia* strains

**DOI:** 10.1186/s12941-024-00691-6

**Published:** 2024-04-10

**Authors:** Chongyang Wu, Li Xiong, Quanfeng Liao, Weili Zhang, Yuling Xiao, Yi Xie

**Affiliations:** Department of Laboratory Medicine, West China Hospital of Sichuan University, Chengdu, Sichuan 610041 China

**Keywords:** *Elizabethkingia*, Antimicrobial susceptibility, Carbapenem resistance, Genomic analysis

## Abstract

**Background:**

*Elizabethkingia* is emerging as an opportunistic pathogen in humans. The aim of this study was to investigate the clinical epidemiology, antimicrobial susceptibility, virulence factors, and genome features of *Elizabethkingia* spp.

**Methods:**

Clinical data from 71 patients who were diagnosed with *Elizabethkingia*-induced pneumonia and bacteremia between August 2019 and September 2021 were analyzed. Whole-genome sequencing was performed on seven isolates, and the results were compared with a dataset of 83 available *Elizabethkingia* genomes. Genomic features, Kyoto Encyclopedia of Genes and Genomes (KEGG) results and clusters of orthologous groups (COGs) were analyzed.

**Results:**

The mean age of the patients was 56.9 ± 20.7 years, and the in-hospital mortality rate was 29.6% (21/71). *Elizabethkingia* strains were obtained mainly from intensive care units (36.6%, 26/71) and emergency departments (32.4%, 23/71). The majority of the strains were isolated from respiratory tract specimens (85.9%, 61/71). All patients had a history of broad-spectrum antimicrobial exposure. Hospitalization for invasive mechanical ventilation or catheter insertion was found to be a risk factor for infection. The isolates displayed a high rate of resistance to cephalosporins and carbapenems, but all were susceptible to minocycline and colistin. Genomic analysis identified five β-lactamase genes (*bla*_GOB_, *bla*_BlaB_, *bla*_CME_, *bla*_OXA_, and *bla*_TEM_) responsible for β-lactam resistance and virulence genes involved in stress adaptation (*ureB/G, katA/B*, and *clpP*), adherence (*groEL, tufA*, and *htpB*) and immune modulation (*gmd, tviB, cps4J, wbtIL, cap8E/D/G*, and *rfbC*). Functional analysis of the COGs revealed that “metabolism” constituted the largest category within the core genome, while “information storage and processing” was predominant in both the accessory and unique genomes. The unique genes in our 7 strains were mostly enriched in KEGG pathways related to microRNAs in cancer, drug resistance (β-lactam and vancomycin), ABC transporters, biological metabolism and biosynthesis, and nucleotide excision repair mechanisms.

**Conclusion:**

The *Elizabethkingia* genus exhibits multidrug resistance and carries carbapenemase genes. This study presents a comparative genomic analysis of *Elizabethkingia*, providing knowledge that facilitates a better understanding of this microorganism.

**Supplementary Information:**

The online version contains supplementary material available at 10.1186/s12941-024-00691-6.

## Background

*Elizabethkingia* spp. are aerobic, Gram-negative bacilli, belonging to the family *Flavobacteriaceae* [1, 2]. Since their discovery in 1959, these microorganisms have been associated with a variety of human infectious diseases, such as meningitis, keratitis and sepsis, that particularly affect immunocompromised individuals [3]. There are currently seven known species, namely, *Elizabethkingia meningoseptica, Elizabethkingia anophelis, Elizabethkingia miricola, Elizabethkingia argenteiflava, Elizabethkingia occulta, Elizabethkingia ursingii, and Elizabethkingia bruuniana*, all of which have undergone various taxonomic and nomenclature changes. In 2003, *E. miricola* was identified for the first time in condensed water samples from the Russian space station Mir [4] and found to be associated with sepsis, bacteremia and pneumonia [5]. In 2011, *E. anophelis* was discovered in the midgut of Anopheles gambiae mosquitos [6] and found to be associated with neonatal meningitis, catheter-associated infections, and two infection outbreaks in Wisconsin with high mortality [6, 7]. In 2018, three new species were redefined, namely, *E. occulta*, *E. ursingii*, and *E. bruuniana* [8]. The number of whole-genome sequences released for *Elizabethkingia* is steadily increasing. Due to their innate resistance to several classes of antibiotics, treatment of *Elizabethkingia* infections is challenging. Consequently, empirical treatment often leads to a relatively high mortality rate. Early diagnosis and prompt initiation of appropriate antibiotics are critical for improving survival rates and outcomes [9]. In addition, several prior studies have reported that patients with a history of persistent antibiotic exposure are more likely to develop nosocomial infections associated with the *Elizabethkingia* genus [10]. Despite the great clinical significance of *Elizabethkingia* infections, gaps remain in our understanding of their demographic characteristics, pathogenicity, and effective treatment options.

*Elizabethkingia* strains have been reported to harbor various types of β-lactamases, which are enzymes responsible for resistance against β-lactams. There are two significant categories of β-lactamases associated with *Elizabethkingia*: class A extended-spectrum-β-lactamases (ESBLs) and class B metallo-β-lactamases (MBLs). The chromosomal genes *bla*_BlaB_, *bla*_GOB_ and *bla*_CME_ are unique to *Elizabethkingia* spp. and contribute to their natural resistance to several commonly used carbapenem antibiotics. The Virulence Factor Database has been employed to predict numerous common virulence factors for *Elizabethkingia.* Many virulence genes have been found to be involved in the synthesis of various components, such as lipo-oligosaccharides, capsule polysaccharides, catalases, proteases, and peroxidases [11]. Additionally, a two-component regulatory system, superoxide dismutase, heat shock protein, and several other factors contributing to the bacterium’s virulence have been identified [12].

## Materials and methods

### Clinical data collection and strains in this study

To accurately investigate the epidemiology and clinical characteristics of *Elizabethkingia* infections, we collected clinical data from 71 patients at West China Hospital of Sichuan University (Chengdu, China) between 2020 and 2021. Seven representative *Elizabethkingia* isolates were routinely collected for further analysis. These isolates were identified using matrix-assisted laser desorption ionization-time of flight mass spectrometry platforms (Bruker Daltonics) with an updated reference spectrum database, and their identities were confirmed via whole-genome sequencing. At the time of the study, there were 83 whole-genome sequences of *Elizabethkingia* species (Supplementary Materials Table S1) available in GenBank (https://www.ncbi.nlm.nih.gov/genome/) (until Dec 01, 2021). All these genome sequences were downloaded for comparative genomic analysis. This database included 43 *E. anophelis*, 16 *E. meningoseptica*, 18 *E. miricola*, 4 *E. ursingii*, and 2 *E. occulta* sequences, which were all isolated from the environment. Among these genomic sequences, 26 were complete genomes, and 57 were shotgun sequences presented as scaffolds or contigs.

### Whole-genome sequencing, assembly and annotation

Seven representative strains were sequenced using an Illumina HiSeq 2500 sequencing platform (Oebiotech, Shanghai, China). Short reads were assembled with SPAdes v.3.6 and optimized based on paired-end and overlap relationships by mapping reads to contigs and scaffolds. The assembled genomes were submitted to the NCBI Prokaryotic Genome Annotation Pipeline and the Rapid Annotations based onProkaryotic Genome Annotation Server (http://rast.nmpdr.org/) for gene function annotation. The annotations were revised using UniProt (http://www.uniprot.org/) and BLAST (https://blast.ncbi.nlm.nih.gov/blast.cgi). Antibiotic resistance genes were identified using the Antibiotic Resistance Genes Database BLAST Server (https://ardb.cbcb.umd.edu/). The Virulence Factor Database (http://www.mgc.ac.cn/VFs/main.htm) was used to identify virulence genes [13].

### Bioinformatics analysis

To ensure uniform and consistent annotations for core and pangenome analyses, all genome sequences were annotated using PROKKA v.1.11 [14]. For genome similarity assessment, average nucleotide identity (ANI) was computed using an ANI calculator (https://www.ezbiocloud.net/tools/ani). Bacterial pangenome analysis (BPGA) [15] was used for comprehensive pan/core genome analysis, including functional annotation of core, accessory and unique genes to cluster of orthologous group (COG) categories and Kyoto Encyclopedia of Genes and Genomes (KEGG) pathways, using the default parameters. A whole-genome sequence-based phylogenetic tree was constructed using the Reference Sequence Alignment Phylogeny builder (REALPHY) [16]. The whole-genome sequences of the 83 *Elizabethkingia* strains were submitted to the REALPHY online pipeline in FASTA format. Sequences acquired in our study and reference sequences downloaded from GenBank were used to construct a phylogenetic tree using PhyML 3.1 [17].

### Antimicrobial susceptibility test

Antimicrobial susceptibility was determined using the VITEK 2 system (bioMérieux, Lyon, France), and breakpoints were interpreted by using the criteria for non-*Enterobacterales* according to CLSI M100-S30 [18]. The minimal inhibitory concentration (MIC) of minocycline was determined by a Kindy-Bauer KB (Thermo Fisher Scientific, USA) following the manufacturer’s recommendations. The breakpoint (MIC > 2 µg/mL) for colistin was determined by the broth microdilution method adapted from the EUCAST [19].

## Results

### Clinical characteristics of *Elizabethkingia* species infections

As shown in Table 1, the specimens were primarily isolated from the respiratory tract (85.9%, 61/71), including sputum (69.1%, 49/71), tracheal secretory fluid (14.1%, 10/71), and bronchoalveolar lavage fluid (2.8%, 2/71). There were only a few samples of sterile body fluids, including cerebrospinal fluid (4.3%, 3/71), ascites (1.4%, 1/71), drainage fluid (2.8%, 2/71), blood (1.4%, 1/71) and pus (2.8%, 2/71). The specimens were predominantly isolated from the intensive care unit (36.6%, 26/71) and emergency (32.4%, 23/71) departments, with fewer specimens from the neurosurgery department (9.9%, 7/71). Most patients had various underlying conditions, such as pulmonary infection, hypertension, and diabetes. A total of 61 patients (85.9%) were hospitalized for more than two weeks. In addition, most patients underwent invasive treatment, including mechanical ventilation (71.8%, 51/71), nasogastric tube placement (63.4%, 45/71) and catheter insertion (59.2%, 42/71). All patients had a history of antibiotic exposure, and 94.4% of patients (67/71) had used broad-spectrum antibiotics for more than one week. The in-hospital mortality rate was 29.6% (21/71).

**Table 1:** Characteristics of 71 patients with *Elizabethkingia* infections

Characteristics	Value
Age(years)	
Rang	–0 ~ 92
–Mean ± SD	56.9 ± 20.7
Gender, n (%)	
–Male	46(64.8)
–Female	25(35.2)
–Hospitalization duration(days), mean ± SD	49.1 ± 40.7
–In-hospital mortality rate, n (%)	21 (29.6%)
Comorbidity, n(%)	
–Hypertension	21(29.6)
–Diabetes mellitus	13(18.3)
–Chronic obstructive pulmonary disease	9(12.7)
–Cardiovascular disease	23(32.4)
–End-stage renel disease	14(19.7)
–Mechanical ventilation, n(%)	51(71.8)
Indwelling device, n(%)	
–Nasogastric tube	45(63.4)
–Urinary catheter	42(59.2)
–Surgical puncture or drain	4(5.6)
Surgery, n(%)	
–Transplantation	1(1.4)
–Chemoradiotherapy, n(%)	1(1.4)
Ward, n (%)	
–Geriatrics	2(2.8)
Nerosurgery	7(9.9)
Orthopaedics	1(1.4)
Intensive care unit	26(36.6)
Neurology	2(2.8)
Emergency	23(32.4)
Urology	1(1.4)
Thoracic surgery	1(1.4)
Cardiac surgery	2(2.8)
Cardiology	3(4.3)
Hematology	1(1.4)
Nephrology	2(2.8)
Side of isolation, n(%)	
Respiratory tract	61(85.9)
Blood	1(1.4)
Urine	1(1.4)
Ascites	1(1.4)
Drainage fluid	2(2.8)
Pus	2(2.8)
Cerebrospinal fluid	3(4.3)

### Antimicrobial susceptibility

Table 2 displays the antimicrobial susceptibility of *Elizabethkingia* isolates and the corresponding MICs for the tested antibiotics. All strains exhibited resistance to 13 ~ 16 antimicrobial agents, including aminoglycosides, macrolides, cephalosporins and carbapenems (imipenem and meropenem). Additionally, they exhibited higher rates of resistance to trimethoprim-sulfamethoxazole. The rate of resistance to piperacillin was 100%, but when piperacillin was combined with β-lactamase inhibitors (cefoperazone/sulbactam and piperacillin/tazobactam), increased sensitivity rates of 90.1% (64/71) and 66.2% (47/71), respectively, were observed. Importantly, all the isolates were found to be susceptible to minocycline and colistin.

**Table 2:** Antimicrobial susceptibilities of 71 *Elizabethkingia* strains

Agents	Resistance (%)	Intermediate (%)	Susceptible (%)
Piperacillin	71 (100)	0	0
Piperacillin-tazobactam	64 (90.1)	1 (1.4)	6 (8.5)
Cefoperazone-sulbactam	47 (66.2)	4 (5.6)	20 (28.2)
Ceftazidime	69 (97.2)	0	2 (2.8)
Ceftriaxone	62 (87.3)	2 (2.8)	7 (9.9)
Cefotaxime	69 (97.2)	2 (2.8)	0
Aztreonam	69 (97.2)	2 (2.8)	0
Imipenem	70 (98.6)	1 (1.4)	0
Meropenem	68 (95.8)	2 (2.8)	1 (1.4)
Gentamicin	68 (95.8)	2 (2.8)	1 (1.4)
Amikacin	68 (95.8)	1 (1.4)	2 (2.8)
Ciprofloxacin	34 (47.9)	5 (7.0)	32 (45.1)
Levofloxacin	25 (35.2)	0	46 (64.9)
Trimethoprim-sulfamethoxazole	26 (36.6)	0	45 (63.4)
Tetracycline	57 (80.3)	10 (14.1)	4 (5.6)
Tigecycline	24 (33.8)	21 (29.6)	26 (36.6)
Minocycline	0	0	71 (100)
Colistin	0	0	71 (100)

### General features of *Elizabethkingia* strains

The next-generation sequencing and assembly data for the 7 genomes are presented in Table 3. The genome size ranged from 4.04 Mb to 4.31 Mb, with an average size of 4.10 Mb, which is consistent with the genome size of the 83 *Elizabethkingia* strains selected from the NCBI database (Supplementary Materials Table S1). The sizes of these 83 genomes ranged from 3.59 Mb to 4.58 Mb, with an average size of 4.26 Mb. The number of contigs per genome ranged from 1 to 378, with a mean of 32.75. The read depth ranged from 23.26 to 80.63, with a mean of 38.79. The average GC content and tRNA content in 83 *Elizabethkingia* strains were 35.84% and 45.42%, respectively.

**Table 3:** General features of seven *Elizabethkingia strains*

Isolate	Specimen	Age	Gender	Whole-genome sequencing	Hospital ward	Length of hospital stay(days)	Size (Mb)	GC %	CDS	Contig	Protein
HX WHF	Cerebrospinal fluid	50	F	*E. miricola*	Neurosurgery	17	4.31	35.83%	4034	63	4079
HX YK	Blood	51	M	*E. anophelis*	Liver surgery	20	4.04	35.61%	3720	70	3764
HX ZCH	Sputum	47	M	*E. anophelis*	Orthopedics	22	4.04	35.61%	3720	76	3764
HX XZB	Cerebrospinal fluid	24	M	*E. miricola*	Emergency	8	4.08	35.81%	3727	43	3769
HX WYD	Blood	92	M	*E. meningoseptica*	Emergency	5	4.08	36.52%	3706	59	3750
HX QKY	Bronchoalveolar lavage fluid	77	M	*E. miricola*	Respiratory medicine	5	4.13	35.83%	3766	59	3813
HX CGY	Blood	89	F	*E. anophelis*	Intensive care unit	10	4.02	35.70%	3706	75	3750

### Resistance-associated genes of the various Elizabethkingia strains

We analyzed the resistance genes of the 83 strains from NCBI and 7 strains from our study and found that different genes were involved in resistance to 6 antibiotics. Antimicrobial resistance genes in the 83 *Elizabethkingia* strains are presented in Supplementary Materials Table S2. These resistance genes included the extended-spectrum β-lactamase genes *bla*_CME,_*bla*_OXA−347_ and *bla*_TEM−116_; the carbapenem resistance genes *bla*_BlaB_ and *bla*_GOB_ and their various subtypes; the aminoglycoside resistance genes *aadS* and *aph(3’)-IIa*; the tetracycline resistance genes *tetX* and *tet36*; and the sulfonamide resistance gene *sul2.* Molecular analysis did not reveal any genes in the mobile colistin resistance (*mcr*) gene family. There was no difference in the distribution of drug resistance genes among the five species. The 7 strains carried all three previously described β-lactamase genes unique to *Elizabethkingia*, including the extended-spectrum β-lactamase *bla*_CME_ and metallo-β-lactams *bla*_BlaB_ and *bla*_GOB_. The specific genes included macrolide, lincosamide and streptogramin (MLS) resistance genes *ermF*, *ereD*, *mefC* and *mphG*. In addition, certain aminoglycoside resistance genes, such as *aac(3)-IVb* and *aac(3)-IIIc*, were only found in our 7 strains (Table S3).

### Virulence-associated genes of the various Elizabethkingia strains

The potential virulence factors and the associated genes of the 83 strains and 7 *Elizabethkingia* strains in our study are shown in Table S2 and Table S4, respectively. We found that some virulence genes, such as catalase/(hydro)peroxidase (*katA*) and translation elongation factor *(tufA)*, were widely distributed in our seven strains. We identified a total of 753 virulence genes in all strains, and 23 kinds of virulence factors could be classified into three types: stress adaptation, adherence and immune modulation. The virulence genes catalase/(hydro)peroxidase (*katA*) and translation elongation factor *(tufA)* were widely distributed in all 90 strains. In addition, the capsular polysaccharide biosynthesis protein (*cps4J*) gene was detected only in *E. miricola*. In addition, 60 K heat shock protein (*htpB*), urease accessory protein (*ureE*), urease beta subunit (*ureB*), Vi polysaccharide biosynthesis (*tviB*) and chaperonin (*groEL*) were identified in these strains. The immune modulation gene *rfbA* and stress survival gene *fcl* were identified only in the 83 strains from the NCBI.

### Core and pangenome analysis and phylogenetic relationships between Elizabethkingia species

**Fig. 1:**
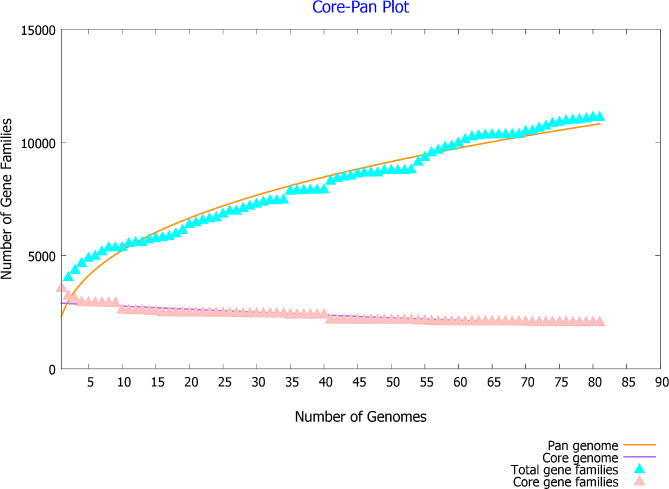
Pan, core and singleton genome evolution according to the number of selected *Elizabethkingia* strains

**Fig. 2:**
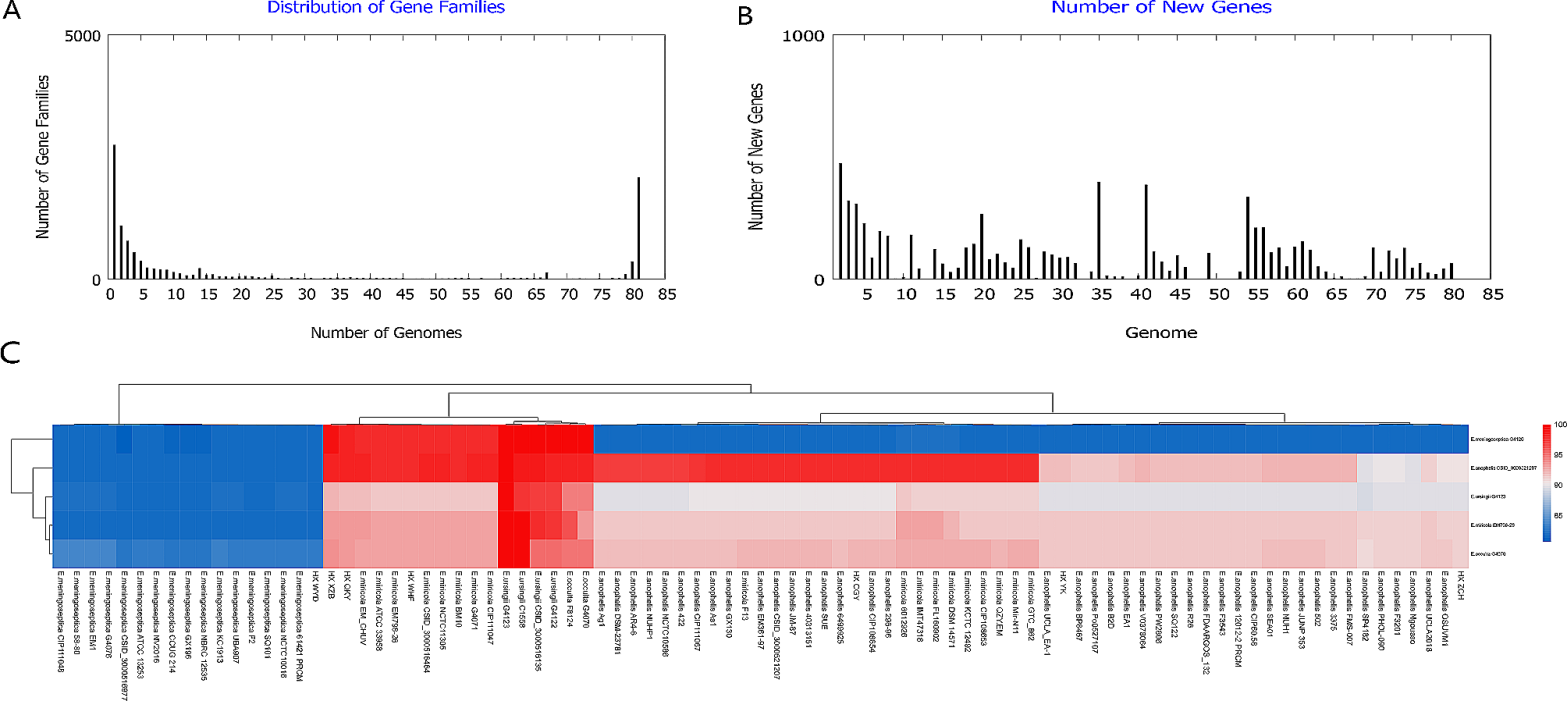
Pangenome analysis of 90 *Elizabethkingia* strains. (**A**) The distribution of the various gene families in the 90 *Elizabethkingia* strains. (**B**) The distribution of new genes in the 90 *Elizabethkingia* strains. (**C**) Dendrogram and heatmap generated using the ANIs of 90 different *Elizabethkingia* strains

To clarify the characteristics and differences in the pangenome between the seven *Elizabethkingia* strains in this study and those from the database, we performed a pangenome analysis on these 90 strains. Core genome analysis revealed that the number of shared genes decreased with the addition of the input genomes. Overall, *E*. *meningoseptica* exhibited an open pangenome feature, with new genes appearing when more sequenced genomes were added to the analysis. Pangenome analysis can be used to determine the diversity of genomes and explore core, accessory, and unique genes. In the 90 strains, 2079 core genes were identified. In each strain, the number of accessory genes ranged from 1097 to 1925, and the number of unique genes ranged from 0 to 364. With the addition of new genome sequences, the number of genes in the pangenome increased from 3265 to 11,813, and the number of core genes decreased from 2546 to 1959 (Fig. 1). The distributions of different gene families and the numbers of new genes are illustrated in Fig. 2A and B, respectively. Whole-genome comparisons allow for the distinction between different strains and species with high resolution. Genome sequences were analyzed using a pairwise method, calculating and comparing the ANI for the 90 *Elizabethkingia* strains (Fig. 2C). The pairwise comparisons revealed a minimum ANI of ∼80.72% for the most distant strains, whereas the *E. anophelis* subspecies showed an ANI of > 98.0%. Additionally, we observed that the ANIs of *E. meningoseptica* and four other species were notably lower than those of the other species. According to the dendrogram, *E. ursingii* and *E. occulta* appeared to be relatively close to *E. miricola*. The delineation of the five species within the *Elizabethkingia* genus was clearly evident in the heatmap generated from the similarity matrix. The phylogenetic analysis of all the isolates was based on whole-genome sequences (Figure S1). Similar to the dendrogram generated from the ANI analysis, *E. ursingii* and *E. occulta* were located close to *E. miricola* and were distant from *E. anophelis* and *E. meningoseptica*. *E. anophelis* was divided into two major sublineages.

### Functional analysis of COGs

**Fig. 3:**
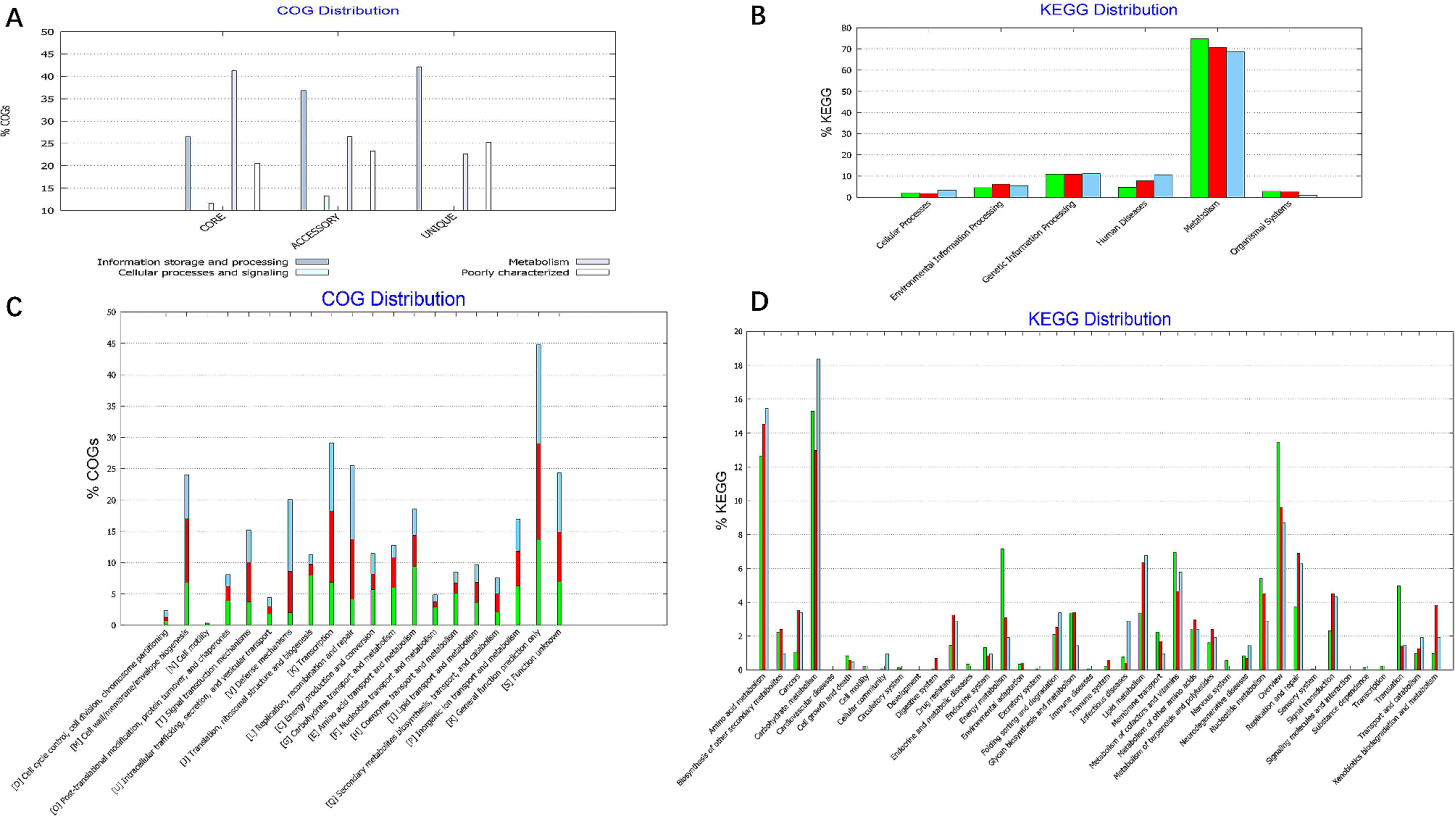
Clusters of orthologous groups (COGs) in the core, accessory, and unique genomes and the associated Kyoto Encyclopedia of Genes and Genomes (KEGG) analysis of 90 *Elizabethkingia* strains. (**A**) Distribution of functional COGs in each core, accessory, and unique genome. (**B**) The detailed distribution of KEGG pathways with their functions. (**C**) The majority of core, accessory, and unique genes were associated with metabolism. (**D**) Functional annotations showing that the gene families associated with carbohydrate metabolism, amino acid metabolism, cofactor and vitamin metabolism, and energy metabolism accounted for the largest proportion of these 90 *Elizabethkingia* strains

COG analysis (Fig. 3A and C) revealed 1,497 conserved genes. There were 1888 accessory genes and 557 unique genes. The functional analysis of the COGs in all the *Elizabethkingia* genomes revealed core, accessory and unique genes related to the regulation of metabolism, cellular processes and signaling, as well as various poorly characterized functions. Core genes were significantly enriched in pathways related to metabolism and biogenesis, including general function, amino acid transport and metabolism, translation, ribosomal structure and biogenesis, and cell wall/membrane/envelope biogenesis. Unique and accessory genes were significantly enriched in transcription; defense mechanisms; and replication, recombination and repair pathways.

The functions of COGs involved in information storage and processing are associated with intracellular survival. In addition, according to the functional prediction of genomes, general function prediction accounted for the largest proportion of COGs, followed by transcription and replication, recombination and repair. Regarding the constituents of each functional gene family, the core genes accounted for the largest proportion of genes related to transcription (11.7%); replication, recombination and repair (10.4%); cell wall, membrane, envelope and biogenesis (7.1%); and defense mechanisms (10.5%).

### KEGG analysis

According to the KEGG analysis (Fig. 3B and D), the largest proportion of genes were enriched in metabolic functions. The other KEGG categories included cellular processes, environmental information processing, genetic information processing, human diseases and organismal systems. The core and accessory genes were most strongly associated with carbohydrate metabolism, followed by amino acid metabolism. Among the genes associated with carbohydrate metabolism, 15.3% were core genes, 12.7% were accessory genes, and 18.5% were unique genes. In addition, the majority of these core genes were also involved in carbohydrate metabolism, and the regulation of amino acid metabolism and lipid metabolism was the focus of the greatest number of genes, followed by signal transduction, replication and repair. In addition to metabolic functions, these genes were also associated with membrane transport, translation, and modulation of cellular growth and death. These functions collectively provide bacteria with the ability to withstand and adapt to the external environment. However, the unique genes in our 7 strains were mostly enriched in KEGG pathways related to microRNAs in cancer, drug resistance (β-lactam and vancomycin), ABC transporters, biological metabolism and biosynthesis, and nucleotide excision repair.

## Discussion

*Elizabethkingia* spp. are responsible for serious nosocomial infections and outbreaks worldwide; however, they have received relatively little attention to date. In this study, we performed whole-genome sequencing and analyzed the characteristics of *Elizabethkingia* strains obtained from clinical samples. This allowed us to perform a comparative genomic analysis of representative clinical isolates alongside other isolates from the NCBI database around the world.

Risk factors for patients with *Elizabethkingia* infections primarily include antibiotic use for more than one week and having three or more underlying diseases. Other risk factors include chronic hemodialysis, multiple injuries, and immunosuppression with prolonged hospitalization. Additionally, in terms of patient diagnosis and treatment, most patients have undergone invasive operations. In this study, mechanical ventilation (71.8%, 51/71), urinary catheter placement (59.2%, 42/71) and nasogastric feeding tube placement (63.4%, 45/71) were identified as the main invasive operations. One of the most significant risk factors for *Elizabethkingia* infection is the use of mechanical ventilation. *Elizabethkingia* can form biofilms in moist environments or on water-related equipment, which facilitates its transmission in hospital settings [20]. The combination of these risk factors and a lack of effective therapeutic regimens can lead to a high mortality rate. In patients infected with *Elizabethkingia*, the mortality rate ranges from ∼20 to 40% [21]. The immune status of patients and virulence factors of microorganisms may be associated with the high fatality rate of patients with *Elizabethkingia* infections. Moreover, numerous previous studies have demonstrated that patients with *Elizabethkingia* infections frequently have chronic illnesses [22–24].

*Elizabethkingia* isolates often exhibit resistance to numerous antimicrobial agents, including β-lactams and inhibitors, aminoglycosides, macrolides, tetracycline, vancomycin, and carbapenems [25]. These strains exhibited variable susceptibilities to piperacillin, piperacillin-tazobactam, fluoroquinolones, minocycline, tigecycline, and trimethoprim-sulfamethoxazole. A wide variety of genes associated with drug resistance in the *Elizabethkingia* genus have been reported. MBLs are a global concern because they can confer resistance against carbapenems and almost all β-lactams. Therefore, evaluating clinical efficacy is crucial since all *Elizabethkingia* spp. appear to be inherent MBL producers [26]. *Elizabethkingia* is the only known organism to carry two distinct MBL chromosomes (*bla*_BlaB_*and bla*_GOB_) as well as the chromosomal *bla*_CME_ gene that can confer resistance to cephalosporins [27, 28]. In addition, comparative analysis revealed that all 90 isolates carried the same subtype of *bla*_BlaB_, and 77.8% (70/71) of the isolates of *Elizabethkingia* spp. carried *bla*_CME−1_. However, various subtypes of the *bla*_GOB_ gene, such as *bla*_GOB−10_, *bla*_GOB−16_, *bla*_GOB−11_, and *bla*_GOB−1_, were identified. Interestingly, genes capable of hydrolyzing cephalosporins, such as *bla*_OXA−347_ and *bla*_TEM−116_, were also identified, which are relatively rare in two *Elizabethkingia* spp. and were not identified in our 7 strains. Regardless of allelic combinations, MIC testing demonstrated that all isolates were highly resistant to carbapenems, penicillin and monobactams. In addition to resistance to carbapenems and β-lactams, several other isolates were found to be resistant to aminoglycosides and macrolides. The resistance genes detected in *Elizabethkingia* spp. included *aadS*, *aph (3’’)-Ia*, *ermF*, *ereD, mef(C)*, and *mph(G)*, and the specific genes *aac(3)-IVb, aph(3’’)-Ia, aac(3)-IIIc*, which conferred aminoglycoside resistance, were identified only in our 7 strains. The presence of multiple resistance genes contributes to the multidrug resistance of *Elizabethkingia* and increases the difficulty of clinical treatment [21]. Based on the results of drug sensitivity tests for this study and previously reported studies [29], the antimicrobial therapy options include minocycline, trimethoprim/sulfamethoxazole, cefoperazone/sulbactam, levofloxacin, colistin or tigecycline, either alone or in combination. Our in-hospital mortality rate of 29.6% (21/71) was lower than that reported in other studies, supporting the value of precision drug combinations in reducing mortality [30, 31].

The potential virulence factor homologs and their associated genes of the five *Elizabethkingia* species were predicted using the VFDB (Table S2). There was no significant difference in the distribution of virulence genes between the two groups of *Elizabethkingia*. These virulence factors include genes involved in capsular polysaccharide biosynthesis, chaperonin, elongation factor, heat shock protein, phospholipase, capsular polysaccharide, catalase, and peroxidase and are responsible for stress survival, immune modulation, and adherence to the environment [32]. Adherence-associated genes, including *tufA*, *htpB* and *groEL*, were widely distributed in all 90 strains, which indicates that these strains have a strong ability to colonize humans or other hosts [33]. In addition, the *fcl* gene encoding the GDP-L-fucose synthetase factor is related to immune modulation [34], but it was not detected in these seven strains. Further studies are necessary to investigate the potential function and source of this putative gene. These candidate genes may constitute promising targets for designing novel strategies to prevent and control infections in species belonging to this highly diverse and environmentally adaptable genus.

*Elizabethkingia* spp. have caused serious nosocomial infections and outbreaks worldwide, and the main aim of this study was to explore the diversity of the clinical characteristics or transmission events of *Elizabethkingia* species isolates from a global dataset. We constructed a phylogenetic tree of 90 *Elizabethkingia* strains based on ANI values, which clearly demonstrated the phylogenetic relationships among these strains. ANI analysis revealed that our seven strains belonged to the same or different species. In addition, comparative genome analysis revealed that the average GC content of *E. meningoseptica* was greater than that of the other four *Elizabethkingia* species, indicating a unique evolutionary pattern and potential receptiveness to mobile genetic elements from other strains.

Pangenome analysis has been used to evaluate genome diversity, genome dynamics, species evolution, pathogenesis and other features of microorganisms [35]. This approach helps us better understand the functional differences among these genes. The gene families of the pangenome represent metabolic capacity, while those of the core genome are typically related to bacterial replication, translation, and maintenance of cellular biogenesis [36]. The core genes are crucial for maintaining cell wall/envelope structures, which shield bacteria from environmental assaults. In our investigation, each strain’s unique genes were widely distributed and associated with functions such as transcription; DNA replication, recombination, and repair; and cell wall/membrane/envelope biogenesis. The unique genes involved in defense mechanisms are subject to relaxed mutation pressure and often correlate with pathogen virulence and pathogenicity. The core genes likely play essential roles in helping these intracellular pathogens survive, regulating cellular metabolism, and mediating cell membrane signal transduction events. However, the unique genes were enriched in poorly characterized functions, suggesting that further research should be conducted to explore their genomic function characteristics. This information might provide useful insights related to clinical infection control and treatment.

## Conclusions

*Elizabethkingia* infections have become a significant public health concern, making it crucial to understand their clinical, molecular, and genetic characteristics. In this study, the complete genome sequences of 90 strains of *Elizabethkingia* were compared and analyzed. The findings contributed new knowledge to our understanding of the population, genome characteristics, and genetic roles of this emerging and life-threatening pathogen. These strains displayed diverse genetic compositions, which might contribute to the particular traits of these genera, as evidenced by the variations in their pangenome sizes.

### Electronic supplementary material

Below is the link to the electronic supplementary material.

Supplementary Material 1

Supplementary Material 2

Supplementary Material 3

Supplementary Material 4

Supplementary Material 5

## Data Availability

The assembled scaffold genome sequences for the seven *Elizabethkingia* species described in this study have been deposited in the NCBI GenBank database under accession numbers SAMN30678013 to SAMN30678019.

## Abbreviations

CLSI: Clinical & Laboratory Standards Institute
EUCAST: The European Committee on Antimicrobial Susceptibility Testing
KEGG: Kyoto Encyclopedia of Genes and Genomes
COGs: Clusters of Orthologous Groups
ANI: Average Nucleotide Identity
ESBL: Extended-Spectrum β-Lactamase
MIC: Minimal Inhibitory Concentration
BPGA: Bacterial Pangenome Analysis
REALPHY: Reference Sequence Alignment Phylogeny Builder
MBLs: Metallo-β-lactamases
NCBI: National Center for Biotechnology Information
BLAST: Basic Local Alignment Search Tool

## References

[1] Kim KK, Kim MK, Lim JH, Park HY, Lee ST (2005). Transfer of Chryseobacterium meningosepticum and Chryseobacterium miricola to Elizabethkingia gen. nov. as Elizabethkingia meningoseptica comb. nov. and Elizabethkingia miricola comb. Nov. Int J Syst Evol MicroBiol.

[2] Bellais S, Aubert D, Naas T, Nordmann P (2000). Molecular and biochemical heterogeneity of class B carbapenem-hydrolyzing beta-lactamases in Chryseobacterium meningosepticum. Antimicrob Agents Chemother.

[3] King EO (1959). Studies on a group of previously unclassified bacteria associated with meningitis in infants. Am J Clin Pathol.

[4] Li Y, Kawamura Y, Fujiwara N, Naka T, Liu H, Huang X (2003). Chryseobacterium miricola sp. nov., a novel species isolated from condensation water of space station Mir. Syst Appl Microbiol.

[5] Zdziarski P, Paściak M, Rogala K, Korzeniowska-Kowal A, Gamian A (2017). Elizabethkingia miricola as an opportunistic oral pathogen associated with superinfectious complications in humoral immunodeficiency: a case report. BMC Infect Dis.

[6] Kämpfer P, Matthews H, Glaeser SP, Martin K, Lodders N, Faye I (2011). Elizabethkingia anophelis sp. nov., isolated from the midgut of the mosquito Anopheles gambiae. Int J Syst Evol Microbiol.

[7] Perrin A, Larsonneur E, Nicholson AC, Edwards DJ, Gundlach KM, Whitney AM (2017). Evolutionary dynamics and genomic features of the Elizabethkingia anophelis 2015 to 2016 Wisconsin outbreak strain. Nat Commun.

[8] Nicholson AC, Gulvik CA, Whitney AM, Humrighouse BW, Graziano J, Emery B (2018). Revisiting the taxonomy of the genus Elizabethkingia using whole-genome sequencing, optical mapping, and MALDI-TOF, along with proposal of three novel Elizabethkingia species: Elizabethkingia bruuniana sp. nov., Elizabethkingia ursingii sp. nov., and Elizabethkingia occulta sp. nov. Antonie Van Leeuwenhoek.

[9] Swami M, Mude P, Kar S, Sarathi S, Mohapatra A, Devi U (2024). Elizabethkingia meningoseptica Outbreak in NICU: an observational study on a Debilitating Neuroinfection in neonates. Pediatr Infect Dis J.

[10] Chiang MH, Chang FJ, Kesavan DK, Vasudevan A, Xu H, Lan KL et al. Proteomic Network of Antibiotic-Induced Outer Membrane Vesicles released by extensively drug-resistant Elizabethkingia anophelis. Microbiol Spectr. 2022:e0026222. 10.1128/spectrum.00262-22 PMC9431301 35852325

[11] Andriyanov PA, Zhurilov PA, Kashina DD, Tutrina AI, Liskova EA, Razheva IV et al. Antimicrobial Resistance and Comparative Genomic Analysis of Elizabethkingia anophelis subsp. Endophytica isolated from raw milk. Antibiot (Basel). 2022;11(5). 10.3390/antibiotics11050648 PMC9137776 35625292

[12] Tang HJ, Lin YT, Chen CC, Chen CW, Lu YC, Ko WC (2021). Molecular characteristics and in vitro effects of antimicrobial combinations on planktonic and biofilm forms of Elizabethkingia anophelis. J Antimicrob Chemother.

[13] Chen L, Yang J, Yu J, Yao Z, Sun L, Shen Y (2005). VFDB: a reference database for bacterial virulence factors. Nucleic Acids Res.

[14] Seemann T (2014). Prokka: rapid prokaryotic genome annotation. Bioinf (Oxford England).

[15] Chaudhari NM, Gupta VK, Dutta C (2016). BPGA- an ultra-fast pan-genome analysis pipeline. Sci Rep.

[16] Bertels F, Silander OK, Pachkov M, Rainey PB, van Nimwegen E (2014). Automated reconstruction of whole-genome phylogenies from short-sequence reads. Mol Biol Evol.

[17] Guindon S, Dufayard JF, Lefort V, Anisimova M, Hordijk W, Gascuel O (2010). New algorithms and methods to estimate maximum-likelihood phylogenies: assessing the performance of PhyML 3.0. Syst Biol.

[18] Clinical and Laboratory Standards Institute (2021). Performance standards for antimicrobial susceptibility testing: 31th informational supplement M100-S31.

[19] The European Committee on Antimicrobial Susceptibility Testing. Breakpoint tables for interpretation of MICs and zone diameters. Version 10.0. 2020. http://www.eucast.org.2020.

[20] Choi MH, Kim M, Jeong SJ, Choi JY, Lee IY, Yong TS (2019). Risk factors for Elizabethkingia Acquisition and clinical characteristics of patients, South Korea. Emerg Infect Dis.

[21] Burnard D, Gore L, Henderson A, Ranasinghe A, Bergh H, Cottrell K et al. Comparative Genomics and Antimicrobial Resistance profiling of Elizabethkingia isolates reveal nosocomial transmission and in Vitro susceptibility to fluoroquinolones, tetracyclines, and Trimethoprim-Sulfamethoxazole. J Clin Microbiol. 2020;58(9). 10.1128/JCM.00730-20 PMC7448627 32580952

[22] Seong H, Kim JH, Kim JH, Lee WJ, Ahn JY. M DN, Risk factors for mortality in patients with Elizabethkingia Infection and the clinical impact of the Antimicrobial susceptibility patterns of Elizabethkingia Species. J Clin Med. 2020;9(5). 10.3390/jcm9051431 PMC7290601 32408478

[23] Tai IC, Liu TP, Chen YJ, Lien RI, Lee CY, Huang YC (2017). Outbreak of Elizabethkingia meningoseptica sepsis with meningitis in a well-baby nursery. J Hosp Infect.

[24] Reed TAN, Watson G, Kheng C, Tan P, Roberts T, Ling CL et al. Elizabethkingia anophelis Infection in Infants, Cambodia, 2012–2018. Emerging infectious diseases. 2020;26(2):320-2. 10.3201/eid2602.190345 PMC6986841 31961289

[25] Lin JN, Lai CH, Yang CH, Huang YH. Elizabethkingia Infections in humans: from Genomics to Clinics. Microorganisms. 2019;7(9). 10.3390/microorganisms7090295 PMC6780780 31466280

[26] Chang TY, Chen HY, Chou YC, Cheng YH, Sun JR (2019). In vitro activities of imipenem, Vancomycin, and rifampicin against clinical Elizabethkingia species producing BlaB and GOB metallo-beta-lactamases. Eur J Clin Microbiol Infect Diseases: Official Publication Eur Soc Clin Microbiol.

[27] Opota O, Diene SM, Bertelli C, Prod’hom G, Eckert P, Greub G (2017). Genome of the carbapenemase-producing clinical isolate Elizabethkingia miricola EM_CHUV and comparative genomics with Elizabethkingia meningoseptica and Elizabethkingia anophelis: evidence for intrinsic multidrug resistance trait of emerging pathogens. Int J Antimicrob Agents.

[28] Wang L, Zhang X, Li D, Hu F, Wang M, Guo Q (2020). Molecular characteristics and Antimicrobial susceptibility profiles of Elizabethkingia Clinical isolates in Shanghai, China. Infect drug Resist.

[29] Lin J-N, Lai C-H, Huang Y-H, Yang C-H. Antimicrobial effects of Minocycline, Tigecycline, Ciprofloxacin, and levofloxacin against Elizabethkingia anophelis using in Vitro Time-kill assays and in vivo zebrafish animal models. Antibiotics. 2021;10(3). 10.3390/antibiotics10030285 PMC7999888 33801839

[30] Rastogi N, Mathur P, Bindra A, Goyal K, Sokhal N, Kumar S (2016). Infections due to Elizabethkingia meningoseptica in critically injured trauma patients: a seven-year study. J Hosp Infect.

[31] Huang YC, Lin YT, Wang FD (2018). Comparison of the therapeutic efficacy of fluoroquinolone and non-fluoroquinolone treatment in patients with Elizabethkingia meningoseptica bacteraemia. Int J Antimicrob Agents.

[32] Chen S, Soehnlen M, Blom J, Terrapon N, Henrissat B, Walker ED (2019). Comparative genomic analyses reveal diverse virulence factors and antimicrobial resistance mechanisms in clinical Elizabethkingia meningoseptica strains. PLoS ONE.

[33] Wang L, Zhang TL, Xiang Q, Fu CX, Qiao M, Ding LJ (2023). Selective enrichment of virulence factor genes in the plastisphere under antibiotic and heavy metal pressures. J Hazard Mater.

[34] Fouts DE, Mongodin EF, Mandrell RE, Miller WG, Rasko DA, Ravel J (2005). Major structural differences and novel potential virulence mechanisms from the genomes of multiple campylobacter species. PLoS Biol.

[35] Guimarães LC, Florczak-Wyspianska J, de Jesus LB, Viana MV, Silva A, Ramos RT (2015). Inside the pan-genome - methods and Software Overview. Curr Genom.

[36] Kumar R, Bröms JE, Sjöstedt A (2020). Exploring the Diversity within the Genus Francisella - An Integrated Pan-genome and Genome-Mining Approach. Front Microbiol.

