# Supplementary material for: Clinical manifestations, antimicrobial resistance and genomic feature analysis of multidrug-resistant Elizabethkingia strains
Source: Ann Clin Microbiol Antimicrob. 2024 Apr 10;23:32. doi: 10.1186/s12941-024-00691-6 (PMC11007976; doi:10.1186/s12941-024-00691-6)
Supplement: Supplementary file 2 — Supplementary Material 2 [file 12941_2024_691_MOESM2_ESM.docx]

Table S4. The potential virulence factor homologs and their associated genes of the seven *Elizabethkingia* strains.

| Classification of Virulence factors | Virulence Factors | YK | CGY | QKY | WYD | WHF | XZB | ZCH |
| --- | --- | --- | --- | --- | --- | --- | --- | --- |
| Stress adaption | Catalase | *katA* | *katA* | *katA* | *katA* | *katA* | *katA* | *katA* |
|  | ATP-dependent Clp protease | *clpP* |  |  | *clpP* | *clpP* |  | *clpP* |
|  | urease beta subunit |  |  | *ureB* |  | *ureB* | *ureB* |  |
|  | urease accessory protein |  |  | *ureG* |  | *ureG* | *ureG* |  |
| Adherence | chaperonin | *groEL* |  | *groEL* | *groEL* |  |  | *groEL* |
|  | heat shock protein |  | *htpB* |  |  | *htpB* | *htpB* |  |
|  | elongation factor | *tufA* | *tufA* | *tufA* | *tufA* | *tufA* | *tufA* | *tufA* |
| Immune modulation | glucose-1-phosphate thymidylyltransferase | *wbtL* | *wbtL* | *wbtL* | *wbtI* |  | *wbtI* | *wbtI* |
|  | GDP-mannose 46-dehydratase |  | *gmd* | *gmd* |  | *gmd* | *gmd* |  |
|  | dTDP-4-dehydrorhamnose 35-epimerase |  | *rfbC* | *rfbC* | *rfbC* |  |  |  |
|  | type 8 capsular polysaccharide synthesis protein |  |  |  | *cap8E, cap8G* | *cap8E* | *cap8D, cap8G* |  |
|  | Vi polysaccharide biosynthesis | *tviB* | *tviB* | *tviB* |  | *tviB* | *tviB* | *tviB* |
|  | capsular polysaccharide biosynthesis protein |  |  |  |  |  | *cps4J* |  |
