# Supplementary material for: Clinical manifestations, antimicrobial resistance and genomic feature analysis of multidrug-resistant Elizabethkingia strains
Source: Ann Clin Microbiol Antimicrob. 2024 Apr 10;23:32. doi: 10.1186/s12941-024-00691-6 (PMC11007976; doi:10.1186/s12941-024-00691-6)
Supplement: Supplementary file 3 — Supplementary Material 3 [file 12941_2024_691_MOESM3_ESM.docx]

Table S3. Antimicrobial resistance genes of the seven *Elizabethkingia* strains.

| Class of antimicrobial | CGY | QKY | ZCH | WHF | | WYD | XZB | YK | |
| --- | --- | --- | --- | --- | --- | --- | --- | --- | --- |
| extended-spectrum β-lactamase | *bla_GOB-9_* | *bla_GOB-11_* | *bla_GOB-10_* | *bla_GOB-10_* | | *bla_GOB-16_* | *bla_GOB-11_* | | *bla_GOB-10_* |
| metallo-β-lactams | *bla_BlaB_, bla_CME-1_* | *bla_BlaB_, bla_CME-1_* | *bla_BlaB_, bla_CME-1_* | *bla_BlaB_, bla_CME-1_* | | *bla_BlaB_, bla_CME-1_* | *bla_BlaB_, bla_CME-1_* | | *bla_BlaB_, bla_CME-1_* |
| aminoglycoside | *aadS,apmA, aph(3'')-Ia,*  *ranA, ranB, mexD* | *ranB,ranA,*  *aac(3)-IVb* | *ranA,ranB,*  *aph(3'')-Ia* | *aadS,erm(A),*  *aph(3'')-Ia, ranA,ranB,*  aadS,  *mphG,ermF* | | *ranA,ranB, aac(3)-IIIc* | *ranA,ranB, aadS* | | *ranA,ranB, aph(3'')-Ia* |
| phenicol | *cmr, cmlv, catB2, catB11* | *catB11* | *catB11* | *catB11* | *catB11* | | *catB11* | | *catB11* |
| macrolide | *ermF, amvA, cfrC, lsaA, muxA* | *lsaA* | *EreD* | *mefC* |  | |  | | *ereD* |
| fluoroquinolones |  | *ceoB* |  |  |  | |  | |  |
