# Supplementary figures and images for: Clinical manifestations, antimicrobial resistance and genomic feature analysis of multidrug-resistant Elizabethkingia strains
Source: Ann Clin Microbiol Antimicrob. 2024 Apr 10;23:32. doi: 10.1186/s12941-024-00691-6 (PMC11007976; doi:10.1186/s12941-024-00691-6)

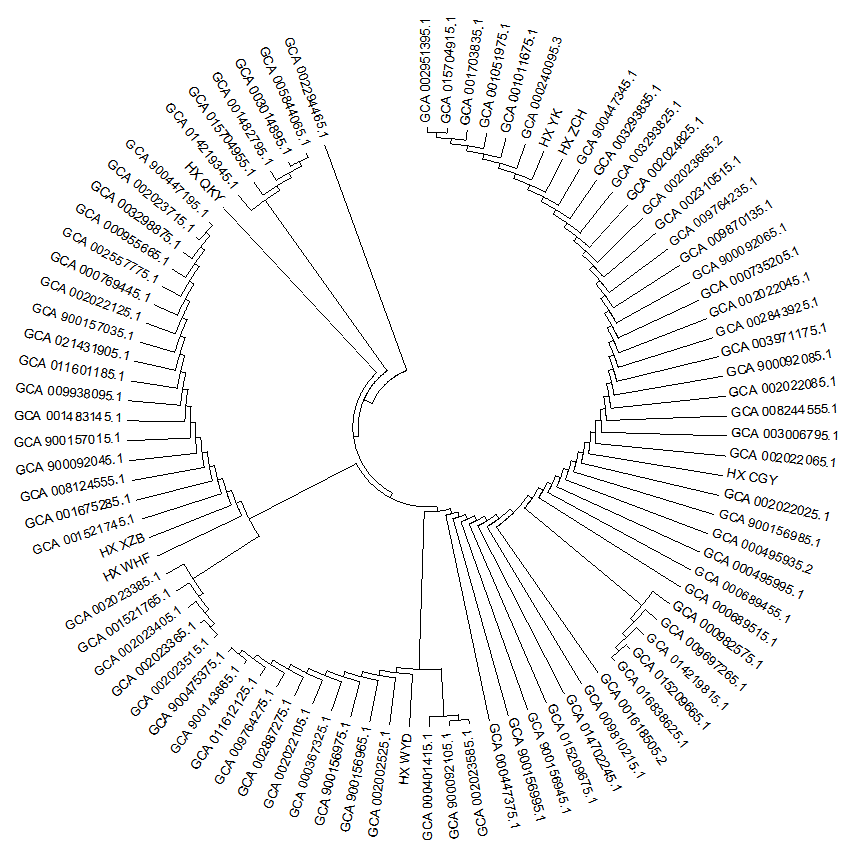

Supplement: Supplementary file 5 — Supplementary Material 5 [file 12941_2024_691_MOESM5_ESM.tif]
